# Quantitative fibre analysis of single-molecule localization microscopy data

**DOI:** 10.1038/s41598-018-28691-5

**Published:** 2018-07-10

**Authors:** Ruby Peters, Juliette Griffié, Garth L. Burn, David J. Williamson, Dylan M. Owen

**Affiliations:** 1 Department of Physics and Randall Division of Cell and Molecular Biophysics, King’s College London, London, UK; 2 Cellular Microbiology, Max Planck Institute for Infection Biology, Berlin, Germany

## Abstract

Single molecule localization microscopy (SMLM) methods produce data in the form of a spatial point pattern (SPP) of all localized emitters. Whilst numerous tools exist to quantify molecular clustering in SPP data, the analysis of fibrous structures has remained understudied. Taking the SMLM localization coordinates as input, we present an algorithm capable of tracing fibrous structures in data generated by SMLM. Based upon a density parameter tracing routine, the algorithm outputs several fibre descriptors, such as number of fibres, length of fibres, area of enclosed regions and locations and angles of fibre branch points. The method is validated in a variety of simulated conditions and experimental data acquired using the image reconstruction by integrating exchangeable single-molecule localization (IRIS) technique. For this, the nanoscale architecture of F-actin at the T cell immunological synapse in both untreated and pharmacologically treated cells, designed to perturb actin structure, was analysed.

## Introduction

Conventional fluorescence microscopy methods produce pixelated images representative of the local distribution of chromophores within the sample, convolved with the microscope point spread function (PSF). This diffraction pattern limits the systems resolving power to ~200 nm, thereby rendering its use limited for the study of cellular ultrastructure on the nanoscale. In recent years, a variety of methods have been adopted to overcome the classical diffraction barrier of light, including SMLM approaches^1–3^. SMLM relies upon the sequential localization of fluorophores, achieved through stochastic activation of sparse subsets of the emitting species. Molecular coordinates are identified by extracting the central position of each temporally separated PSF, routinely achieving 10–30 nm lateral localization precision. SMLM data takes the form of a list of molecular coordinates each with an estimated localization uncertainty; a SPP. Localization-based method of super-resolution, therefore embrace a mathematical framework for data analysis.

Quantification of clustered SPPs generated from SMLM data is now routinely used to extract biologically relevant information regarding protein nanoscale organization^4–7^ and has previously provided insight into cellular nano-organisation and function^8–10^. However, analysis of SPPs of a fibrous nature remains comparatively understudied. We have previously shown that an angular version of Ripley’s K-function can provide overview statistics on fibrous SPPs including an assessment of fibre mesh density, fibre linearity and orientational regularity^11^ and Nieuwenhuizen *et al*. have demonstrated a tool to quantify fibre co-orientation^12^. Tracing of fibres has been demonstrated, albeit by sacrificing the pointillist nature of the data and rendering as a conventional image^13^ and the field of principle curve tracing through scattered x-y data is well established in statistics. Nevertheless, to our knowledge, no method exists to accurately trace fibrous structures through pointillist SMLM data sets and extract quantitative information from those traces. Directly analysing the fundamental pointillist data is an important advance. Whilst there are numerous methods for rendering pixelated images from pointillist data, there is no ideal method to do this, and no consensus on the best approach^14^. Thus, pixilation introduces variability on the derived image meaning that such an input into a fibre tracing algorithm is further from the ground truth. Additionally, this variability makes the comparison of tracing algorithms that use pixelated data as input intrinsically difficult as they perform differently with pixelated images with different statistical composition and construction. Analysing the fundamental, pointillist data removes this layer of variability ensuring high fidelity, un-manipulated input data with well-known statistical properties.

The method presented here assigns a density parameter to each point through Voronoi tessellation^4,15^ segmenting events without the need for radial or toroidal based scanning, thereby removing topological bias. The algorithm then traces a path that minimises this density parameter gradient, constrained by radial and angular restrictions, to create a landscape of fibrous structures. This fibre landscape can then be interrogated to determine fibre characteristics such as number of fibres, fibre lengths and enclosed meshwork areas. In addition, it can extract points of fibre branching and characteristic branch point angles. We demonstrate, using various simulated pointillist data sets, that the proposed method is capable of accurately extracting these fibre descriptors.

There are many examples in which the characterisation of nanoscale fibrous structures is important, and, additionally, imaging of the actin and tubulin cytoskeletons is frequently used to demonstrate super-resolution microscopy systems. One notable example is the picket-fence model^16,17^ first proposed by Kusumi *et al*. which now has strong experimental and theoretical support^18–20^. Here, the nanoscale structure of the cortical actin meshwork is responsible for modulating membrane protein diffusion and clustering, with important implications for cell signalling. We apply the methodology to the complex meshwork of filamentous (F-) actin at the T cell immunological synapse. It is known that F-actin forms a dense meshwork at the synapse periphery^21^ which flows inwards in a retrograde fashion^22,23^ – this dense mesh is pseudo-2-dimensional in its architecture and is only accessible using super-resolution techniques^24,25^. Indeed, in other immune cell synapses, both structured illumination microscopy (SIM) and stimulated emission depletion (STED) have been used to extract descriptors of the actin cytoskeletal mesh, where it was shown that remodelling of the mesh was important for vesicle trafficking and release^26,27^. We characterise the fibrous mesh properties at the synapse periphery and synapse centre and show that the actin disrupting agent Cytochalasin D results in fewer, shorter fibres and therefore fewer enclosed regions.

## Results

### Description of algorithm

**Figure 1:**
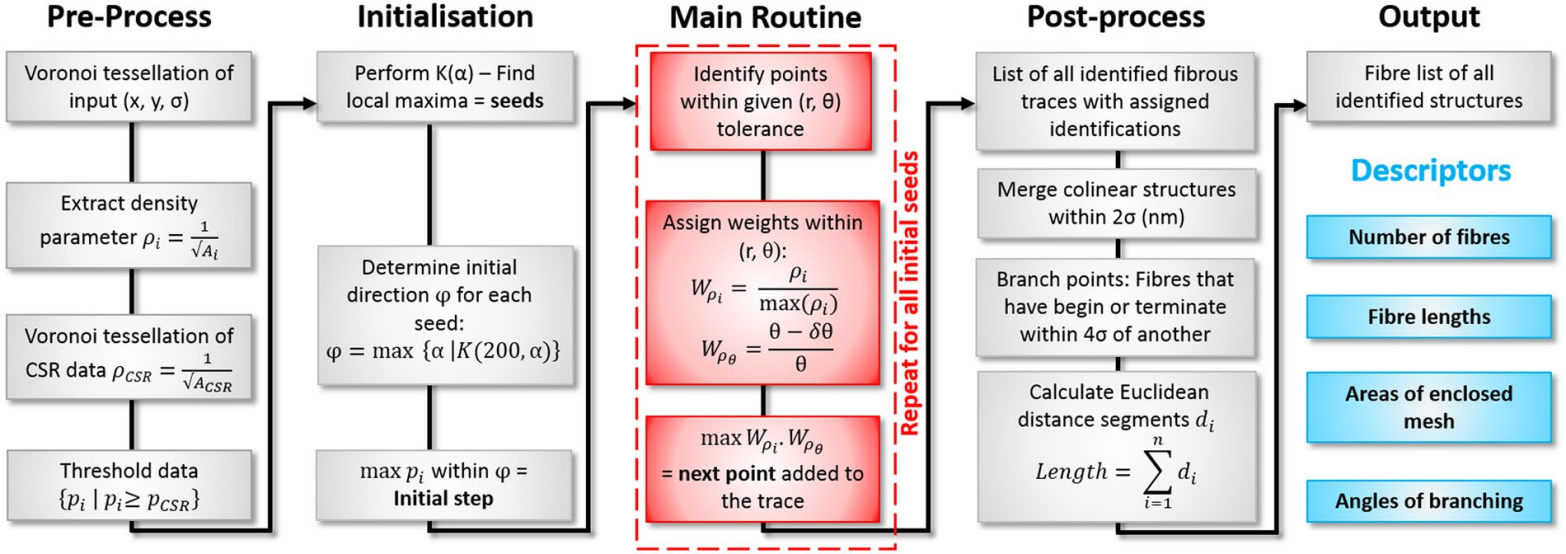
Representative workflow for the proposed fibre analysis of SMLM data.

The algorithm is based upon a fibre tracing routine that seeks to minimise its weighted density parameter gradient as localizations are assigned to a fibre. The method takes, as input, the coordinates of all localizations generated by localization software (Thunder-STORM in our case). We begin by performing a Voronoi tessellation of the localized data, from which a density parameter can be calculated per ${p}_{i}=\frac{1}{\surd {A}_{i}}$, where A_i_ is the area of the i^th^ Voronoi cell. All points are then subject to a threshold, such that only those with density parameters above that of an equivalent distribution of simulated completely spatially random (CSR) points remain^28,29^. As per our previous method, the modulation depth of the angular Ripley’s K-function^30^ indicates the points possessing the most fibrous nature^11^. Using local maxima – fibrous points with high modulation depth as seeds for the fibre tracing routine assures that, for each fibre, the trace begins at the most fibrous location. To initialize the trace, all localizations within a distance r (nm) are identified. To determine the initial direction of the fibre *φ*, we extract the most fibrous angular range from the seed point – as calculated by the Ripley’s angular K-function^11^. The localization with the highest density parameter in this direction is chosen as the initial step for the trace. Using this vector as the initial direction, we search for more localizations to add to the growing fibre trace. Given a fixed radius and angle tolerance, points that are identified along the fibre direction (averaged over the two previous steps) are assessed. Localizations are attributed to the fibre if they have the highest weighted density ${W}_{{p}_{i}}$ and angle ${W}_{{p}_{\theta }}$ parameters out of all the assessed localizations. For this, we weight each localization’s density parameter by that of the maximum identified within the search region and calculate the angular disparity between the absolute vector of the fibre direction θ and the vector δθ of the query localization. The highest product of the two weighted parameters provides the next localization to be added to the trace. This process is repeated until there are no more points to consider in the fibre direction. At this stage, the tracer returns to the absolute maximum, the initial seed, and the main routine is repeated in the opposite (−180°) direction. This process is reiterated for all local maxima until a list of identified structures is generated. Post-processing of the fibre list further concatenates structures according to both collinearity and local distance constraints, to provide the complete fibre list. The output of the algorithm is a fibre landscape, containing traces of all identified structures, from which several descriptors of fibre architecture can be obtained. Using the points assigned to each fibre, one can directly calculate fibre length, number of fibres, number of branch points and branching angles. A summation of the analysis workflow is presented in Fig. 1. Conceptually, the algorithm attempts to trace a path along “ridges” radiating from local maxima by taking the next step which minimises its change in weighted density parameter.

### Simulated results

We tested the method using a variety of simulated fibrous point patterns. To represent experimental conditions, possible labelling positions were simulated every 3.4 nm along each fibre, within a square (3 × 3 μm) region of interest (ROI). For each position, the extent of sampling (labelling) was varied, to represent non-uniform fluorophore labelling and possible multiple blinking. 30% of positions received no label, 40% one label, 25% two labels, with the remaining 5% of positions receiving three to six labels. To simulate localization uncertainty, all points were disturbed by Gaussian noise, the mean and variance of which was determined by a Gamma distribution with shape, k, and scale, θ, parameters determined such that the mean simulated localization uncertainty was 16 nm, matching our experimental data. The entire point pattern was then overlaid with a CSR background of non-fibrous localizations, totalling 30% of the total localizations. We refer to such parameters as the Standard Condition.

**Figure 2:**
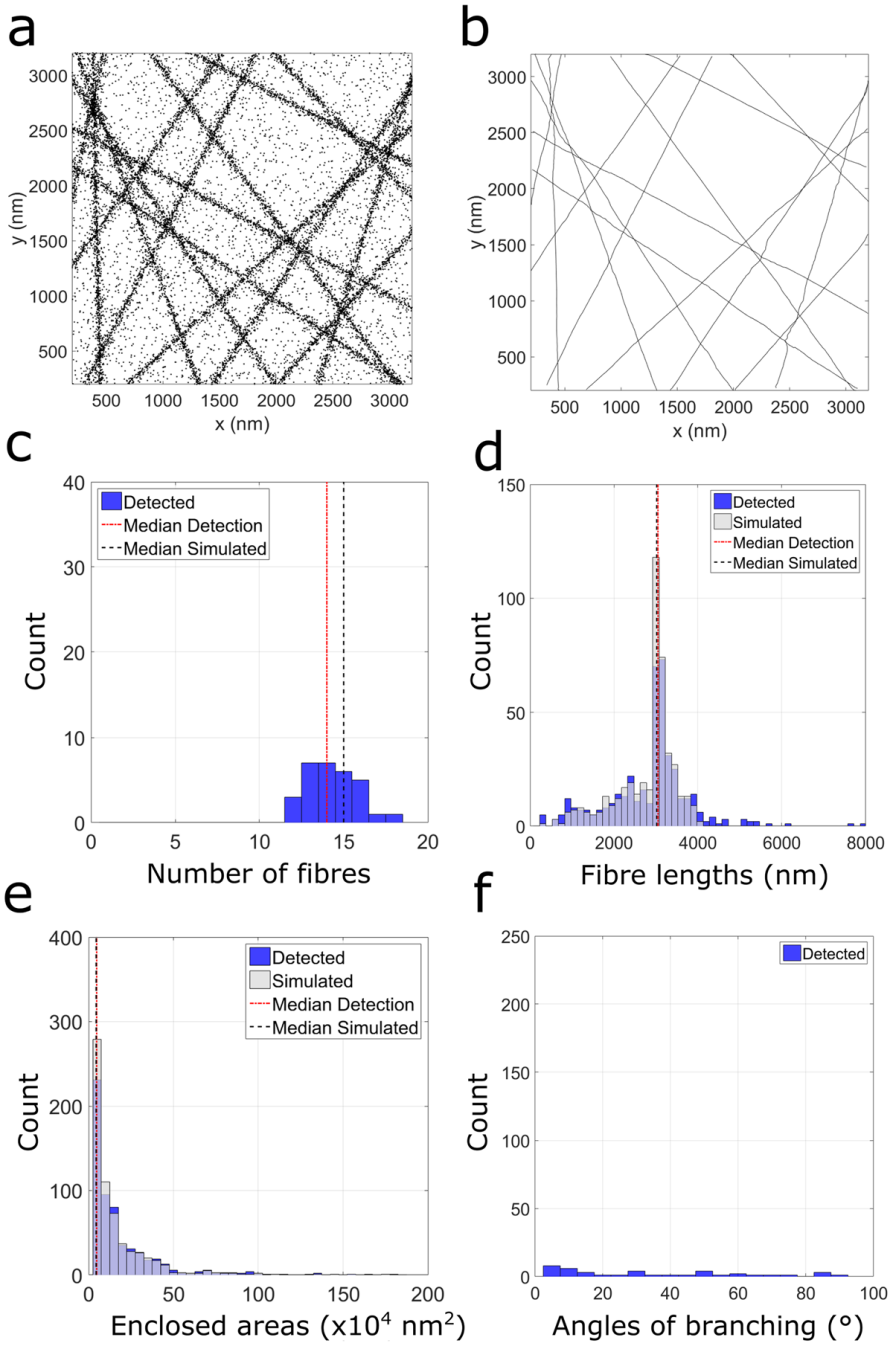
Fibre analysis (n = 30 simulations) of 15 randomly distributed linear fibrous structures generated per the Standard Condition. A representative fibrous point pattern (**a**) and the corresponding output (**b**) of fibre tracing. Histograms of number of fibres (**c**) fibre lengths (**d**) areas of enclosed regions (**e**) and angles of identified branching points (**f**).

We begin by considering the case of a simulated meshwork of 15 fibres placed with random directionality across the ROI, otherwise generated as per the Standard Condition. A representative input (Fig. 2a) and output (Fig. 2b) of the tracing routine (from n = 30 simulations), demonstrates that the method is capable of tracing heterogenous fibre orientations within the ROI. Histograms (n = 30 simulations) of four key fibre descriptors – number of fibres (Fig. 2c), fibre lengths (Fig. 2d), area of enclosed mesh (Fig. 2e) and angles of identified branching points (Fig. 2f) – showed close alignment with simulated values. Note that various other descriptors such as a measure of curvature, eccentricity of mesh areas, and centroids of enclosed areas can be further extracted from the complete fibre lists. Moreover, the method can provide information on the global properties of the fibrous point pattern. A notable result includes the distribution of enclosed areas (Fig. 2e), which demonstrates a lack of regularity in mesh organisation. This finding is evidenced by the narrow distribution of enclosed areas determined for a regularly arranged (400 nm) fibrous SPP (Supplementary Fig. 1). We further tested the method against the curved analogue of this simulated scenario (Supplementary Fig. 2). For this, we choose at random either clockwise or anticlockwise arcs of known curvature (R = 8000 nm) and generate the fibres as per the Standard Condition. Fibre descriptors (from n = 30 simulations) indicate that the method can accurately trace this type of fibre architecture. To test the validity of the method to trace curved structures, a measure of curvature was extracted (Supplementary Fig. 3), defined as the ratio of the detected fibre length to the Euclidean start-end distance, per fibre. To evaluate the methods capability of tracing fibres that do not span the entire ROI, we also simulated point patterns of shorter fibres at two different fibre densities (Supplementary Figs 4 and 5). The algorithm detects fibre descriptors in both cases. To further test the capability of the method, we preformed simulations of a reduced and inconsistent labelling density. For this, points were deleted at random from the standard condition data set, up to 95% deletion (Supplementary Fig. 6). Results indicate that the method is largely insensitive to this parameter, up to 60% deletion, after which fibres become prematurely terminated. The effect of an increased average localization uncertainty on the analysis was also investigated (Supplementary Fig. 7). The method remains accurate up to ~40 nm uncertainty in localization, and as such is suitable for techniques such as d-STORM (direct-Stochastic Optical Reconstruction Microscopy).

**Figure 3:**
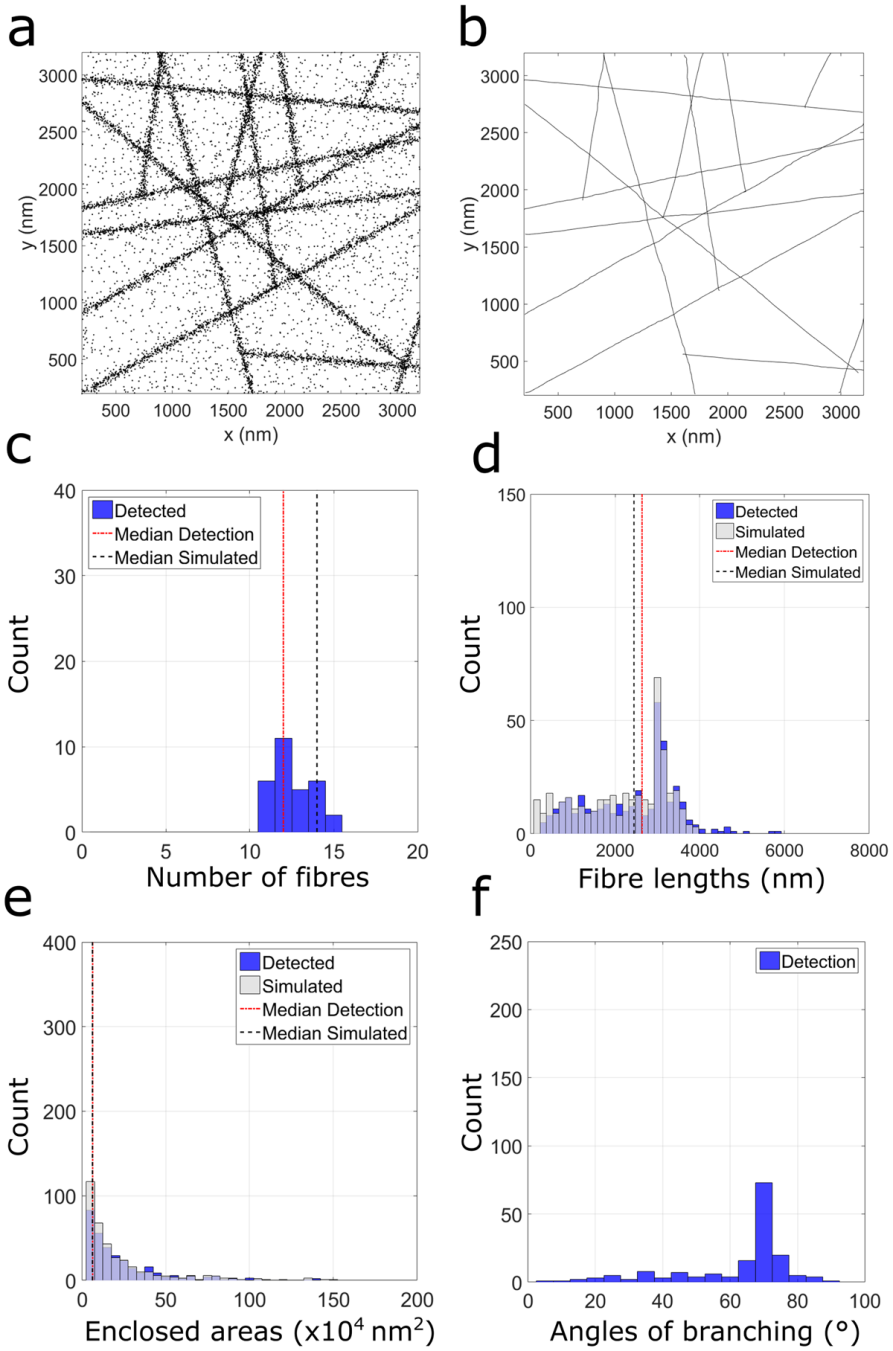
Fibre analysis (n = 30 simulated) of a branched fibrous network generated using the Standard Condition. A representative input (**a**) of localizations and resulting fibre landscape (**b**) output. Histograms of number of fibres (**c**) fibre lengths (**d**) areas of enclosed regions (**e**) and angles of identified branching points (**f**).

Branched actin fibres form part of the dense actin network in many biological systems. The Arp 2/3 complex plays an important role in the regulation of actin polymerisation, serving as a nucleation site for the growth of daughter filaments orientated 70° to the mother filament^31^. We therefore tested the methods capability of detecting such an arrangement of complex fibre architecture. For this, we begin by simulating 7 mother filaments, from each of which one daughter filament is generated (at a random location) at 70°. A representative (from n = 30 simulations) input (Fig. 3a) and resultant fibre landscape (Fig. 3b) demonstrates that the algorithm can accurately extract information regarding the branched fibrous meshwork. In this case, the distribution of fibre lengths (Fig. 3d) closely aligns with that of the simulated values, despite localization uncertainty and noise. Further, the area of enclosed regions (Fig. 3e) indicates that the underlying fibre network is not regularly arranged. The final descriptor, the angle of detected branching points (Fig. 3f) clearly exhibits a peak branching angle of 70°, as per simulation. For this detection, we consider a branching fibre as one whose start or end lies within a distance 4 times greater than that of the mean localization precision (~60 nm) of any other fibre, excluding ROI edges (Post-processing, Fig. 1).

### Experimental results

We next demonstrate our method using experimental data. T cell activation is critical for the development of the adaptive immune response. Upon encountering antigen presenting cells (APCs) displaying antigenic peptides on surface Major Histocompatibility Complex (MHC), CD4+ Helper T cells form a junction known as the immunological synapse^32^. Actin fibres form a pseudo-2-dimensional dense meshwork or largely tangential fibres at the mature synapse periphery^22,25^, with the central regions forming a sparser, radial distribution^11^. To facilitate SMLM, we formed artificial synapses between the Jurkat T cell line and an activating glass coverslip coated with anti-CD3 and anti-CD28 antibodies (See Methods)^10^. Cells were fixed and labelled using the actin-binding peptide LifeAct coupled to the dye Atto655N. The repeated and transient binding of this peptide with F-actin allows PSF localization with extremely high localization precisions and labelling densities^33^. Mature (7 mins post-activation) T cell synapses were imaged under total internal reflection (TIRF) illumination, and PSFs localized using the ThunderSTORM plugin to ImageJ^34^. 3 × 3 μm regions were cropped from either central or peripheral regions and analysed. As a further demonstration, cells were treated with Cytochalasin D before fixation, which disrupts polymerised actin fibres.

**Figure 4:**
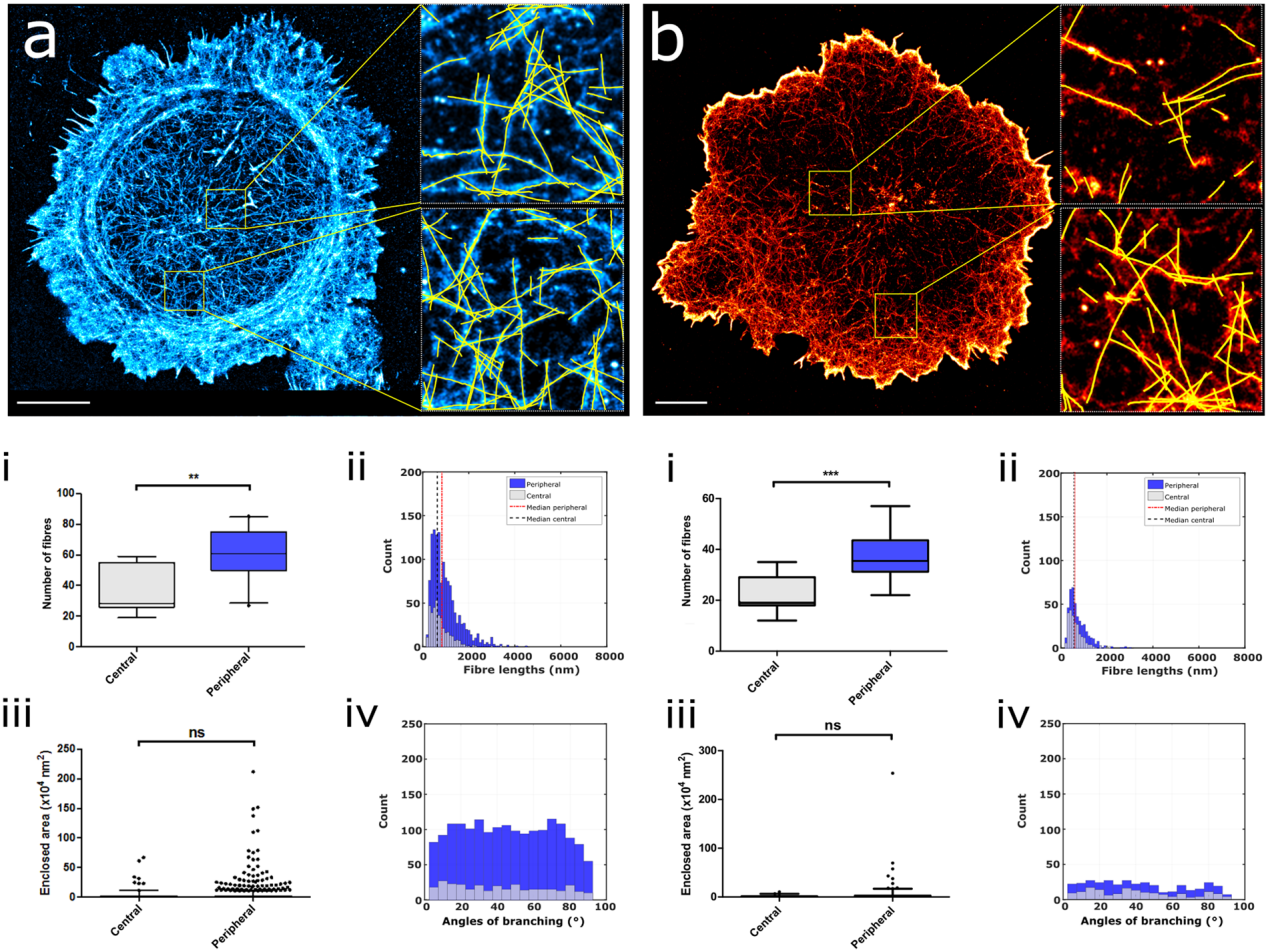
IRIS imaging of the F-actin meshwork in T cell synapses. Representative IRIS images of a mature T cell synapse (Panel a) and a Cytochalasin D treated synapse (Panel b) with inset fibre analysis examples of 3 × 3 μm regions. Quantitation of untreated cells in both central and peripheral regions are shown for both control (n = 5 cells, n = 15 regions, (**a**,i–iv)) and actin disrupting (n = 3 cells, n = 15 regions, (**b**,i–iv)) conditions. Scale bar 5 μm. ns = not significant, *p < 0.05, **p < 0.005, ***p < 0.0005.

A representative reconstructed SMLM image taken under control (untreated) conditions is shown (Fig. 4a) together with selected and analysed regions from the central and peripheral regions. Extracted fibre descriptors from a total of n = 15 central and peripheral regions are shown in Fig. 4a,i–iv. The data indicate a statistically significant doubling of the fibre density from approximately 30 fibres per ROI to 60 (p < 0.005). Fibres in the periphery were also significantly longer than in the centre (p < 0.0005). Together, these longer, more numerous peripheral fibres enclosed more regions than in the centre, although these regions were the same size. Although no single characteristic 2D branch angle could be detected, the total number of branch angles was also higher in the peripheral regions (p < 0.0005). Figure 4b shows the equivalent data for Cytochalasin D treated cells. While the key trends between the synapse centre and its periphery remain, compared to controls, treated cells exhibit significantly fewer fibres (p < 0.05 for central, p < 0.0005 for peripheral regions), and those that are present are shorter in length (p < 0.0005 for both central and peripheral regions). The short length of fibres, coupled with their sparsity, mean that very few regions are enclosed, and very few branch points are detected. Interestingly, pooling of all selected regions from both central and peripheral zones enables a global understanding of cytoskeletal change upon pharmacological treatment. Globally, there are fewer fibres in Cytochalasin D treated cells (p < 0.0005), which were significantly shorter (p < 0.0005). The approximate level of curvature for control cells was also evaluated (Supplementary Fig. 3) and indicates that within the ROI constraints, the F-actin filaments exhibit a linear architecture.

Despite the method being applied to IRIS data of T cell synapses, the method is fully applicable to other localization based methods of super-resolution microscopy which generate SPPs. To demonstrate this, we performed dSTORM imaging of the microtubule network in a fixed HeLa cell (see Methods), which exhibited an average localization uncertainty of ~26 nm. The method is successful in identifying fibrous structures, in both dense and sparse regions (Supplementary Fig. 8).

## Discussion

Unlike conventional fluorescence microscopy, SMLM allows the determination of the position of fluorescent molecules within the sample with a precision ten times higher than the classical diffraction limit. The data generated by this new form of microscopy is a SPP, no longer a pixeled image. A challenge for researchers is to interrogate these SPPs to extract meaningful and quantitative information about the underlying biological structures. In the simplest case, this might involve determining whether detected fluorescent molecules are randomly arranged within the sample, or, contrary, form clustered distributions. Many varied tools now exist to this end^5–7^. A relatively unexplored area of analysis however concerns the quantification of fibrous SPPs derived from SMLM. This is particularly important because firstly, the architectures of the many cytoskeletal components are crucially important in determining cell behaviour, including signalling. This is nowhere better illustrated than the picket fence model of Kusumi, which has extensive theoretical and experimental support^15,18,20,35^. Secondly, because cytoskeletal components are a frequent test-bed for developments in SMLM labelling, hardware and localization technology and are thus ubiquitous in the field. Here, we have demonstrated a fibre analogue of widely-used pointillist cluster analysis algorithms.

Based on measurements of local density and angular constraints, we demonstrate that we can accurately trace fibres through pointillist SMLM data. Validated using simulations, we show that such properties as the number of fibres, length of fibres, areas enclosed by fibres as well as positions and angles of branch points can be extracted from the data, without any need to render a conventional pixelated image. Indeed, the multifarious methods of SMLM data rendering provide different images, and a comparison between approaches is not quantitively evaluated^14^. Several parameters contribute to achieving accurate visualisation of SMLM data, from convolution methods to sampling criterion. As such, by exploiting the localization co-ordinates as input alone for data analysis, one can ensure a fairer comparison not only between conditions but across all SMLM data. The method is tested by characterising the cortical actin meshwork at the T cell immunological synapse^22,32^, where we demonstrate that F-actin at the synapse periphery consists of denser, longer fibres and that, as expected, Cytochalasin D treatment results in a sparser meshwork of shorter fibres.

Here, the experimental synapse data was acquired via IRIS – a transient binding approach to SMLM which yields extremely high labelling density and localization precision^33^. However, the method is fully applicable to other SMLM methodology such as DNA PAINT^36^, dSTORM^37^ or PALM^1^. The biological system used here also presents the simplifying property of being a pseudo-2-dimensional actin meshwork capable of being fully imaged using TIRF illumination. As was the case with the first applications of cluster analysis in this field^5^, which also assumed 2D geometry, the development of 3D fibre tracing algorithms will surely be an important future development as seen with 3D cluster analysis^29,38^. Also important, will be the development of multi-colour fibre analysis and cluster-fibre co-analysis mirroring initial advances into two-colour co-cluster analysis^39,40^. Like cluster analysis, fibre analysis of SMLM data has the potential to elucidate many fundamental aspects of cell nanoarchitecture and its effect on cell behaviour, with implications for understanding human health and disease.

## Methods

### Cell culture and synapse formation

Jurkat E6.1 T cells (ATCC TIB-152) were maintained in Roswell Park Memorial Institute (RPMI-1640) medium (+10% fetal bovine serum (FBS)). Prior to plating, glass coverslips coated with anti-CD3 (clone OKT3) and anti-CD28 (clone 28.2) antibodies (Cambridge Bioscience and BD Bioscience, UK) at a concentration of 2 mg/mL and 5 mg/mL respectively, were thermally equilibrated. For F-actin disruption studies synapses were engineered as above and treated with 0.1 µM Cytochalasin D (Sigma-Aldrich, US). Cells were activated on the stimulatory coverslip for 7 minutes at 37 °C to allow mature synapse formation. Surplus cell suspension was removed, and a fixation buffer added (10 mM MES (pH 6.1), 138 mM KCl, 2 mM EGTA, 320 mM sucrose, 3 mM MgCl pH. 7.0 + 4% para-formaldehyde (PFA)) for 15 minutes at 37 °C. Post-fixation, coverslips were washed in phosphate buffered saline (PBS) x3 followed by permeabilization in 0.1% Triton X-100 for 5 minutes at 4 °C. Following additional washes, coverslips were blocked using a buffer consisting of 50 mM NH_4_Cl, 10 mM Glycine, 2% (w/v) Fish Skin Gelatin (FSG), 2% (w/v) bovine serum albumin (BSA), for 30 minutes at room temperature (RT). Additional washes were performed prior to the addition of the LifeAct-Atto655N IRIS probe at 0.5 nM.

### HeLa preparation

HeLa cells were cultured in Dulbecco’s Modified Eagle Medium (DMEM) supplemented with 5% FBS. Prior to imaging, cells were seeded at a density of 50 × 10^3^ cells/cm^2^ on a chambered coverslip (ibidi µ-slide 8 well) and left overnight at 37 °C (+5% CO_2_). Growth media was removed and immediately replaced with pre-warmed fixation solution comprising of paraformaldehyde (3.2% (v/v)) in PEM buffer: 80 mM PIPES, 5 mM EGTA, 2 mM MgCl_2_, pH 6.9 and kept at 37 °C for 15 minutes. Post-fixation, cells were washed x2 with pre-warmed PEM buffer for 5 minutes at 37 °C, followed by permeabilisation with 0.01% (w/v) lysolecithin (LPC) (in PEM buffer) at RT for 10 minutes. Permeablisation solution was removed and the sample was washed x3 in tris-buffered saline (TBS). A block/quench buffer (TBS pH 7.6, 2% BSA (w/v), 0.2% FSG (w/v), 50 mM ammonium chloride, 10 mM glycine) was added for 10 minutes at RT followed by a blocking buffer (TBS pH 7.6 containing 2% BSA (w/v), 0.2% FSG (w/v)) for 1 hour at RT. Samples were then incubated with an anti-α-tubulin antibody (clone DM1A, at 1 µg/mL in TBS + 1% BSA (w/v)) overnight at 4 °C, followed by 3 × 5 minute washes in TBST (TBS + 0.1% Tween-20 (v/v)). A goat-anti mouse secondary antibody conjugated to AlexaFluor 647 was then added to the sample for 1 hour at RT at 2 µg/mL (in TBS + 1% BSA (w/v)). Cells were then washed 3 × 5 minutes in TBST, followed by a single wash in TBS. The chambers were then filled with a dSTORM imaging buffer (base buffer consisting of 0.56 M glucose, 50 mM Tris (pH 8.5) and 10 mM NaCl supplemented with 5 U/mL pyranose oxidase, 10 mM cysteamine (Sigma M6500), 40 µg/mL catalase (Sigma C100) and 2 mM cyclooctatetraene (Sigma 138924). Coverslips were overfilled and sealed with parafilm to minimise oxygen penetration into the buffer solution.

### SMLM Imaging

All SMLM imaging was performed using the commercial N-STORM 4.0 system (Nikon) operated in TIRF mode through a 100 × 1.49 NA oil-immersion lens. Cells were imaged using a 647 nm excitation laser (~1.125 kW/cm^2^) and fluorescence captured on a EMCCD camera iXon3 DU-897E (Andor Technology Ltd.) at 25 °C. A quad bandpass emission filter set (Chroma 89902-ET-405/488/561/647 nm) was used to filter the fluorescence collected from the sample. Images were collected at 50 ms and 20 ms integration time for IRIS and dSTORM imaging respectively, and a total of 100,000 frames were typically recorded for image reconstruction.

### Image Reconstruction

Reconstructions were generated using the ImageJ plug-in ThunderSTORM^34^. Single molecule localizations were estimated using the local maximum approach with 8-neighbourhood connectivity. PSF models were fitted for sub-pixel localization using the integrated Gaussian method. Post-processing included removing localizations with uncertainties (calculated per Thompson *et al*.)^41^ greater than 15 nm (for IRIS images), drift correction via cross correlation and finally merging of molecules detected in more than 20 consecutive frames within a radius of 20 nm.

### Statistical analysis

Two-tailed non-parametric t-tests were calculated using 95% confidence intervals using Prism software.

## Electronic supplementary material

Supplementary Information
