# Supplementary material for: Quantitative fibre analysis of single-molecule localization microscopy data
Source: Sci Rep. 2018 Jul 10;8:10418. doi: 10.1038/s41598-018-28691-5 (PMC6039472; doi:10.1038/s41598-018-28691-5)
Supplement: Supplementary file 1 — Supplementary Information [file 41598_2018_28691_MOESM1_ESM.pdf]

Quantitative fibre analysis of single-molecule localization microscopy data: Supplementary material

Ruby Peters[1], Juliette Griffié[1], Garth L. Burn[2], David J. Williamson[1] and Dylan M. Owen[1*]*

[1] Department of Physics and Randall Division of Cell and Molecular Biophysics, King's College London, London, UK.

[2] Cellular Microbiology, Max Planck Institute for Infection Biology, Berlin, Germany.

* To whom correspondence should be addressed: ruby.peters@kcl.ac.uk

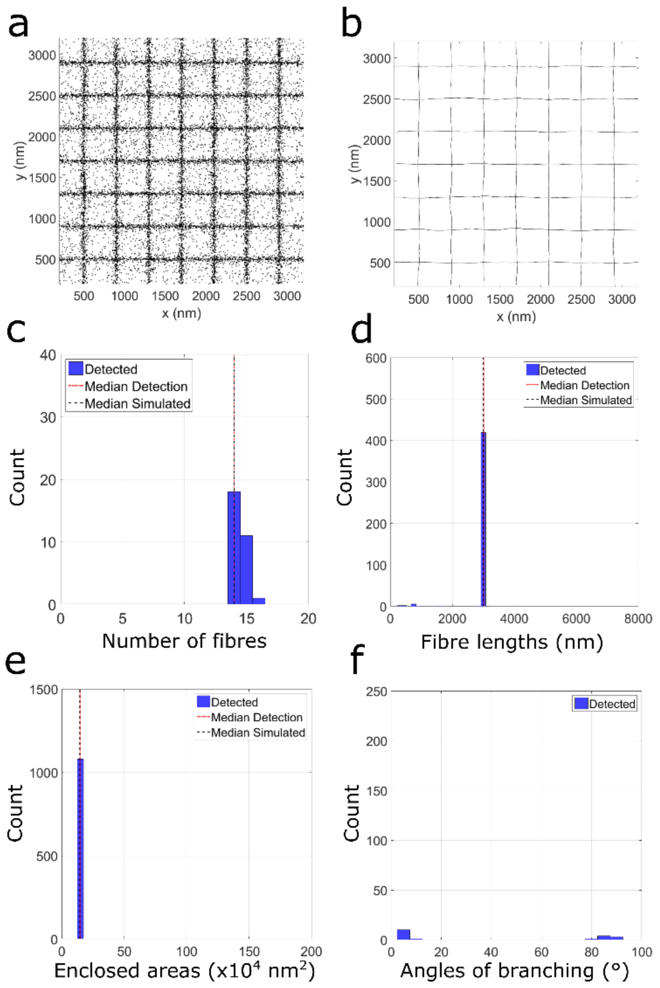

Supplementary Figure 1. Fibre analysis (n=30 simulated) of regular fibrous spatial point patterns generated using the Standard Condition. A representative input (a) of localizations and resulting fibre landscape (b) output. Histograms of number of fibres (c), fibre lengths (d), areas of enclosed regions (e) and angles of identified branching points (f)

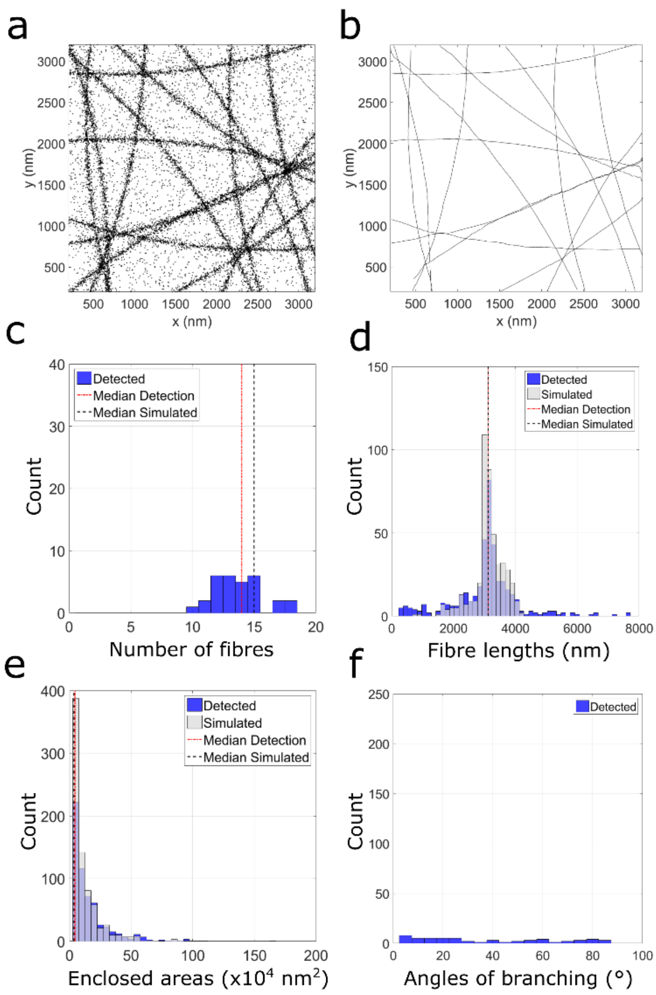

Supplementary Figure 2. Fibre analysis (n=30 simulations) of 15 randomly distributed fibrous structures with curvature generated per the Standard Condition. A representative fibrous input (a) and the corresponding output (b) of fibre tracing. Histograms of number of fibres (c), fibre lengths (d), areas of enclosed regions (e) and angles of identified branching points (f).

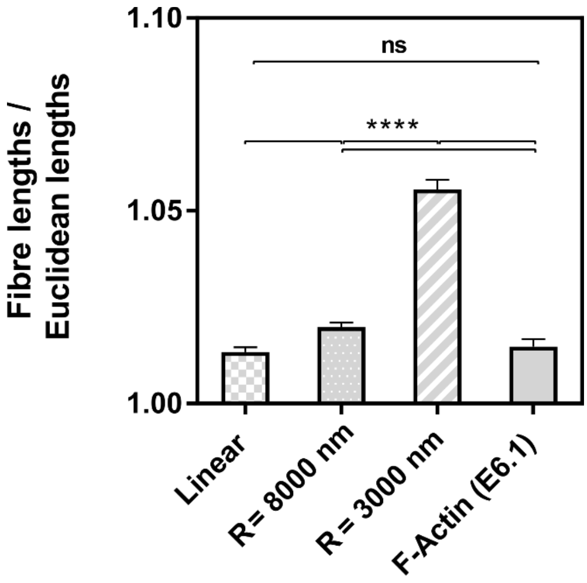

Supplementary Figure 3. Approximate curvature of detected fibres for the a linear, curved (R= 8000 nm) and more curved (R=3000 nm) simulation sets (n=30) and control (E6.1) T cell synapses. Error bars represent the 5-95% CI of the median. ns = not significant, **** = $p < 0.0001$.

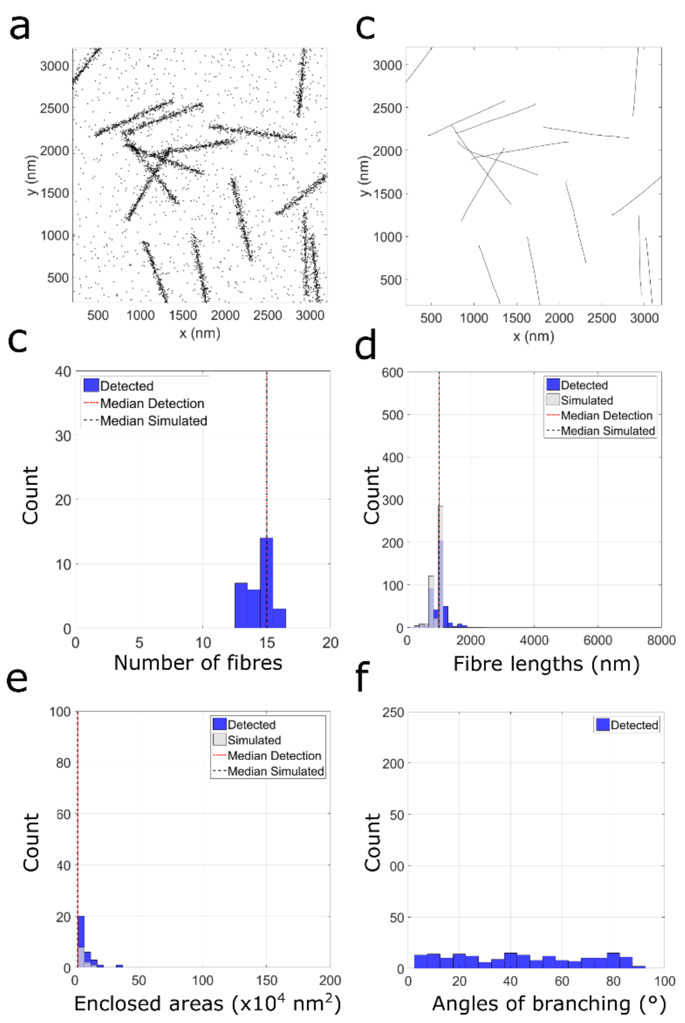

Supplementary Figure 4. Fibre analysis (n=30 simulated) of 15 fibres of 1µm maximum length randomly placed within the region, generated using the Standard Condition. A representative input (a) of localisations and resulting fibrous (b) output. Histograms of number of fibres (c), fibre lengths (d), areas of enclosed regions (e) and angles of identified branching points (f).

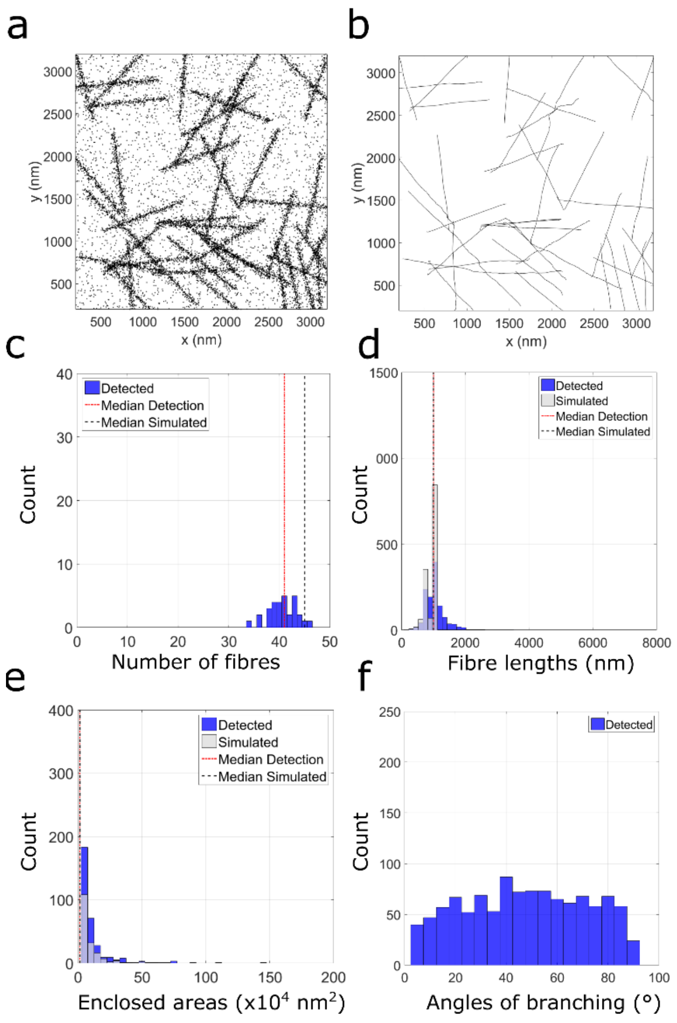

Supplementary Figure 5. Fibre analysis (n=30 simulated) of 45 fibres of 1µm maximum length randomly placed within the region, generated using the Standard Condition. A representative input (a) of localisations and resulting fibrous (b) output. Histograms of number of fibres (c), fibre lengths (d), areas of enclosed regions (e) and angles of identified branching points (f).

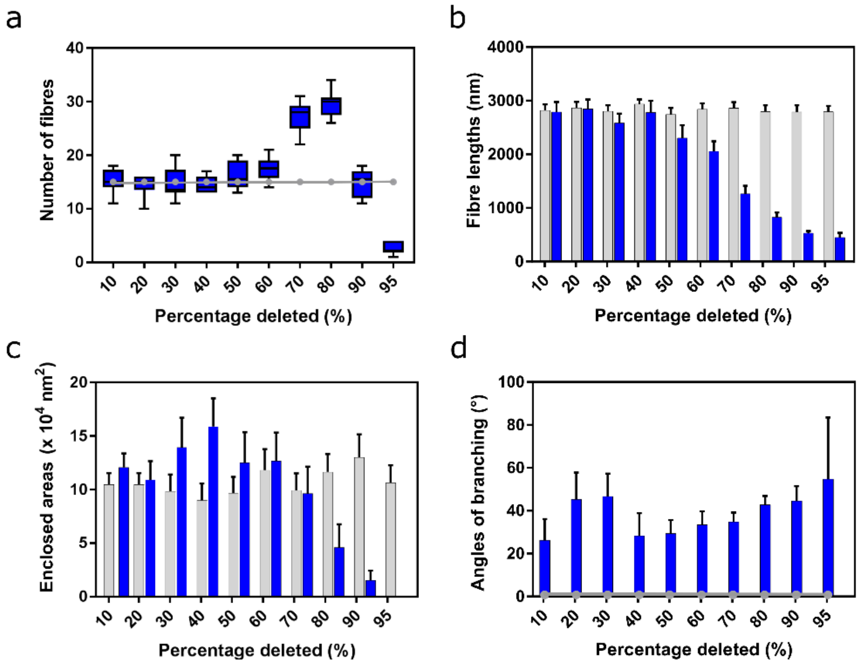

Supplementary Figure 6. Examining the effect of reduced and inconsistent labelling density of target structures on the analysis method. The number of fibres detected (a), the lengths (b), areas of enclosed regions (c) and angles of branching points (d) versus the percentage of points deleted from the standard condition data set. For all cases, detected and simulated values are depicted blue and grey respectively. Error bars represent 5-95% CI of the median for n=10 simulations per condition.

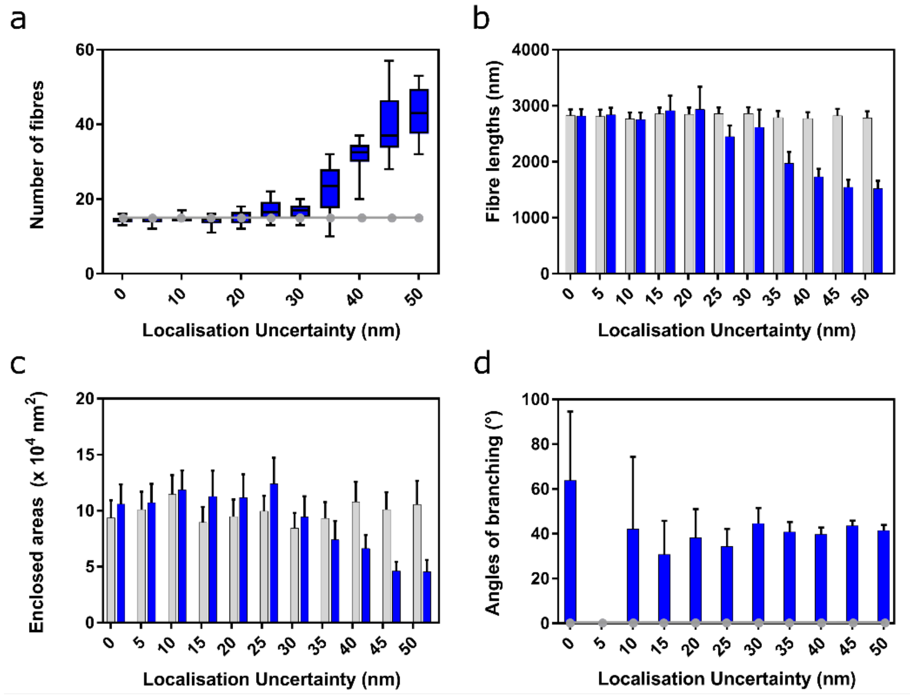

Supplementary Figure 7. Examining the effect of an increased localisation uncertainty in the SPP on the analysis method. The number of fibres detected (a), the lengths (b), areas of enclosed regions (c) and angles of branching points (d) versus the average simulated uncertainty values. For all cases, detected and simulated values are depicted blue and grey respectively. Error bars represent 5-95% CI of the median for n=10 simulations per condition.

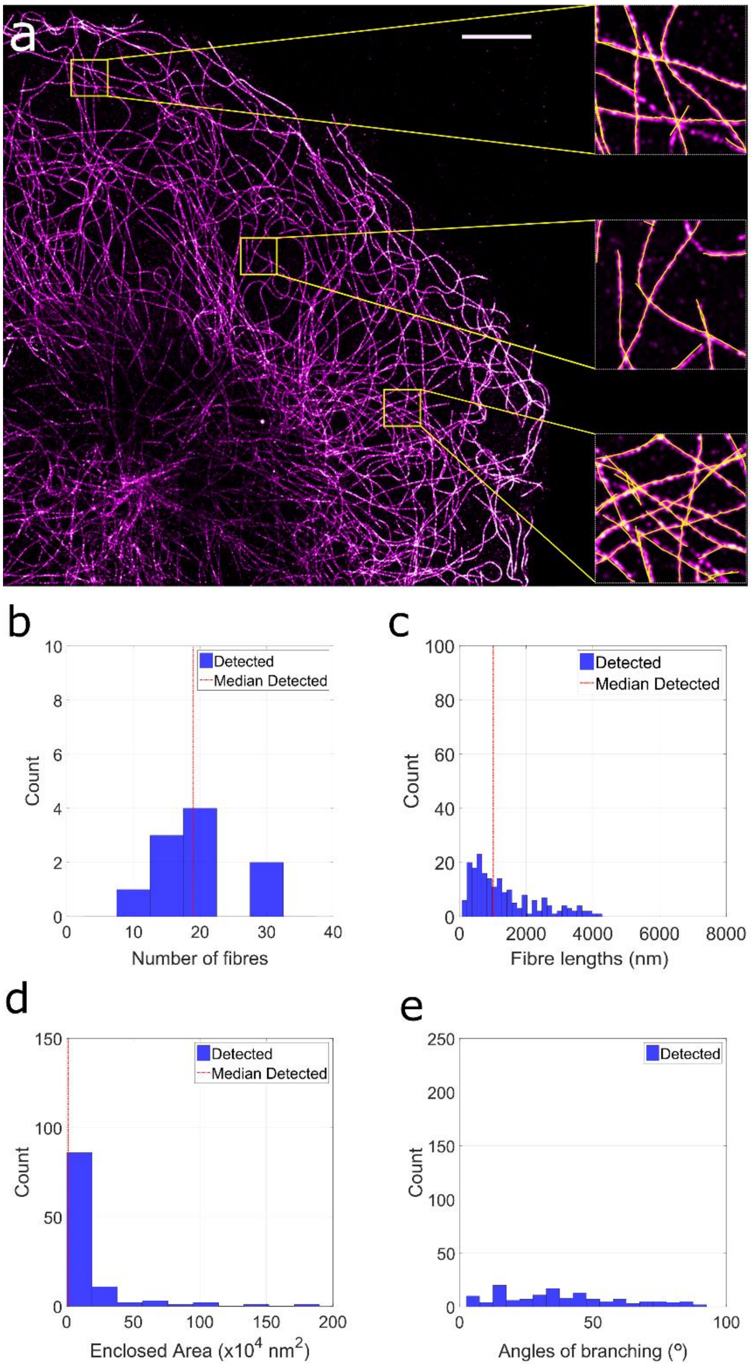

Supplementary Figure 8. Fibre analysis of a microtubule network in a fixed HeLa cell. A reconstructed cell (a) is depicted with inset exemplar regions (3 x 3 µm), onto which fibre traces are shown (yellow). The number of fibres detected (b), the lengths (c), areas of enclosed regions (d) and angles of branching points (e) are presented for n=10 ROIS. Scale bar 5µM.
